# Realising the Power of Academic-Voluntary Sector Partnerships to Integrated Care Research

**DOI:** 10.5334/ijic.9101

**Published:** 2025-05-13

**Authors:** Kelly Hall, Darshini Ayton, Helen Skouteris, Robin Miller, Catherine Needham

**Affiliations:** 1 University of Birmingham, UK; 2 Monash University, Australia

**Keywords:** Voluntary, community and social enterprise organisations (VCSEs), health and social care, research, co-production

## Abstract

Voluntary Community and Social Enterprise organisations (VCSEs) bring valuable knowledge of local health and care needs and have skills in engaging underserved communities. This makes them ideal partners for integrated health and care researchers. Yet, they are often excluded from research. This paper reflects on the benefits and challenges of VCSE-researcher collaborations and shares examples from Australia and the UK of how these have been overcome in practice. We explore how integrated care researchers can work with VCSEs to empower the voice of lived experience, bring greater inclusivity in research methodologies and deliver meaningful findings within local and diverse contexts.

## Introduction

Voluntary Community and Social Enterprise organisations (VCSEs) are vital providers of health and care services in countries like the UK and Australia. A key goal of VCSEs is to reduce health inequalities by supporting diverse communities, often those experiencing the most vulnerability, to improve health and care outcomes. They are increasingly recognised as community experts and representatives with valuable knowledge of local health and care needs and skills in engaging underserved communities. This reflects the increasing focus on population health and inclusive practice within integrated care [1].

Evaluating the impacts of integrated care requires a ‘multifaceted, methodologically robust approach’ [2, P1], which draws insights from data and perspectives held by different stakeholders. Research therefore needs to address implementation processes and challenges within a local context to avoid findings simply highlighting broad truisms, and for people with lived experience to be at the heart of research design, including those from underserved communities. VCSEs, with their community connections, embeddedness within local contexts, and user-focussed principles, are therefore ideal partners for integrated health and care researchers [3]. Yet, VCSEs working with communities facing health and care inequities are often excluded from research. Subsequently, implementation science theories, models, and frameworks that we use to address the gap between scientific knowledge and its application in practice, known as the research-to-practice gap, have been developed mostly void of input from those with lived experience of the problems being addressed, including VCSEs [4].

This perspective piece is written by research teams in Australia and the United Kingdom who have worked extensively with VCSEs in relation to health and care research. We reflect on the considerable opportunities that VCSE-researcher collaborations bring, the challenges experienced and provide two examples of how these have been overcome in practice. We conclude with our learning on future actions within integrated care research which could make the most of these potential opportunities.

## The Benefits and Challenges of VCSE-Researcher Partnerships

Many research funders require researchers to demonstrate meaningful collaboration with public and practitioner partners. Collaborations between VCSEs and academics are an important part of this landscape, as VCSEs can bring expertise around how to engage with communities, especially under-represented communities, to co-produce innovations that matter to them. This can deliver research that is more tailored, accessible and meaningful and deliver positive benefits to people and communities through changes to practice and policy. In turn, VCSEs benefit from being close to research, shaping priorities, and rapidly incorporating learning into practice.

Despite these benefits, our extensive experience shows there are barriers to meaningful collaboration between academic researchers and VCSEs, including that researchers are not always aware of the public contributors, lived experience experts and interest-holders that exist within their research ecosystem nor of the best way to engage them in equal partnerships. Conversely, VCSEs and lived experience experts are not always aware of the significant role they can play in research and how they can be empowered to use their expertise to generate research ideas, co-develop research solutions, interpret findings, and co-author on research papers/reports. This leaves many researchers and VCSEs not knowing where to start or how to develop equitable partnerships with each other. In Australia and the UK, we have developed initiatives to address these challenges and overcome the research-to-practice gap.

## Creating Equitable Research Partnerships: Research Better Together

VCSEs are often engaged in academic research through consultation rather than co-creation, leading to unequal power relationships. A project in the UK called ‘Research Better Together’ has sought to redress this power imbalance and facilitate the positive engagement of VCSEs in integrated care research. Co-designed between academics at the University of Birmingham, VCSEs and National Health Service partners, Research Better Together [5] seeks to build sustainable and equitable VCSE-researcher partnerships, that also include communities traditionally underserved by research. It provides opportunities for VCSEs and researchers to create joint research agendas, through networking events and shared learning opportunities.

Equitable partnerships can also be hindered by research funding systems, which rarely pay VCSEs and lived experience experts to fully engage at the design stage. Academic timelines and processes, including research ethics and methodologies, can also be unfamiliar to VCSEs. In response to these challenges, Research Better Together provides small grants to researcher-VCSE partnerships to fund the time of VCSEs and the communities they support, to co-design projects from the outset. Training on research systems (including ethics and funding) is also provided, along with mentorship to build the partnership and agree ways of working. An example of a partnership involved an academic, two VCSEs and a public health sector partner co-designing a research project to evaluate the role of health and wellbeing coaching within underserved communities. Research Better Together funding was used to pay for the time of VCSE contributors during the research design phase, along with lived experience experts from underserved communities who were involved as ‘community researchers’. With training and mentorship on research funding offered through Research Better Together, this equitable VCSE-academic-community partnership have now gone on to secure a sizeable research grant.

## Addressing the research-practice gap: Researcher in Residence

In Australia, the Government prioritises bi-directional mobility and trusted collaboration to build skills and share knowledge between VCSEs and researchers. However, it has long been recognised that researchers and VCSE practitioners operate in two distinct worlds: the world where researchers produce knowledge and the world where this research is used. Over the last decade, a ‘researcher in residence’ approach has been adopted to facilitate rapid knowledge exchange and to bridge the research-practice gap. Monash University’s Health and Social Care Unit have embedded researchers in residence across VCSEs in health, social care, and education for over eight years. Their researchers reside at their host organisation for part of their appointment. This means making themselves available for, and involving themselves in, the organisation’s day-to-day operations. As researchers in residence, they are afforded real-time working knowledge of the needs, interests, pain points and experiences of the organisations they aim to support through their research. This enables them to create and translate knowledge that answers the research and practice questions of their host organisation, breaking down hierarchies and siloes by ensuring researchers are not seen as the “elite experts” who hold PhD qualifications but rather as genuine partners working in collaboration [6].

For example, one of their researchers in residence collaborated with a social enterprise, bestchance Child Family Care, to co-develop the Social-Emotional Engagement and Development (SEED) program with parents, educators, and health professionals. SEED shifts the research paradigm to prioritise the needs of the early childhood education and care sectors. They addressed the challenges identified by educators in supporting children’s social and emotional learning (SEL) by co-developing the first public health approach to SEL to promote young children’s mental health through tiered layers of SEL intervention, partnerships between teachers, families and allied health professionals, and investment and resourcing at the organisational and policy level [7]. SEED, now owned by bestchance and funded by the Victorian Department of Education, is implemented across 109 early years services, benefiting over 300 educators and 3,500 children.

## Learning for Future Integrated Health and Care Research

For integrated health and care research to contribute to sustained, systematic change, it will need to involve people and communities to ensure a focus on topics that are important to them and different perspectives are included [3]. The role of VCSEs in facilitating and brokering this form of involvement is crucial but has not always been well understood or supported through sufficient investment and on-going support. There are mutual benefits for researchers and VCSEs working together, and each bring distinct strengths. Researchers often have access to external research grant funding and bring a formality and independence to research design which can facilitate more objective findings and greater credibility of impacts by government and funding bodies. VCSEs have connections and skills that enable more creative ways of working together in genuinely participatory and emancipatory ways. As our above examples demonstrate, realising the potential of VCSE-research collaborations is possible with flexible approaches that respect the different contributions of each partner and seek to build long-term, sustained relationships. Going forward, there should also be more opportunities for VSCE practitioners to have hybrid roles in practice and research, like those commonly open to health professionals, and to access training and support in integrated health and care research methodologies. To be successful, integrated care requires a diversity of voices and influence – this is as true of its research as its practice.
